# Veratrum Viride

**Published:** 1867

**Authors:** 


*Veratrum Viride.*

[Dr. Ira Hatch, of this city, has suggested the following correction, which is due to the memory of that eminent teacher, Dr. Tully.—Editor]

Dr. Percy, in his admirable prize essay, makes a mistake, which, in justice to Dr. Tully's memory, should be corrected. Speaking of the medicinal properties of veratrum viride, Dr. Percy says:—"About the year 1830, Drs. Tully and Ives, of New Haven, investigated, very fully, the therapeutic properties of the plant. Is veratrum viride narcotic? Tully and Osgood state that it is." By reference to Tully's Materia Medica, it will be seen that he investigated its properties as early as 1810. It will also be seen, that he had, later in life, fully come to the conclusion that it was not narcotic, whatever he might have taught in his lectures, previous to the date of Dr. Osgood's "very truthful and praiseworthy essay." On pp. 727–8 of his Materia Medica, Dr. Tully remarks that:—"The term *acrid narcotic*, as employed by authors, has reference as much to such articles as veratrum viride, etc., as to any article in which an individual, active, proximate principle is at one and the same time acrid and, as is supposed, narcotic. But do veratrum viride, sanguinaria, etc., possess any true and genuine narcotic power? Do they ever prove directly anti-irritant, anodyne, and soporific? Is not such an operation essential to a true and genuine narcotic?"

"Although I once entertained a different opinion, (I believe mainly on the ground of authority,) yet I am now obliged to doubt whether veratrum viride or sanguinaria ever prove directly anti-irritant, anodyne, or soporific. For myself, I have not the least doubt that they are non-narcotic erethistics. I have been in the habit of employing both of them ever since the summer of 1810, and I never have witnessed anything like a direct 'narcotic'* effect from either of them, though I have often witnessed erethism, when they have been given in comparatively large doses, at short and regular intervals."

* The word narcotic is substituted for anti-irritant, etc.

